# Capitalising on the transformational opportunities of early clinical academic career training for nurses, midwives and allied health professionals

**DOI:** 10.1186/s12909-020-02348-2

**Published:** 2020-11-10

**Authors:** Alison Cowley, Claire Diver, Alison Edgley, Joanne Cooper

**Affiliations:** 1 Nottingham University Hospitals NHS Trust, Institute of Care Excellence, Derwent House, City Campus, Hucknall Road, Nottingham, NG5 1PB UK; 2 School of Health Sciences, University of Nottingham, Queen’s Medical Centre, Nottingham, NG7 2HA UK

**Keywords:** Allied health professionals, Clinical academic careers, Education, Evidence-based practice, Nurses, Midwives, Transformational leadership

## Abstract

**Background:**

A highly skilled workforce is required to deliver high quality evidence-based care. Clinical academic career training programmes have been developed to build capacity and capabilities of nurses, midwives and allied health professionals (NMAHPs) but it remains unclear how these skills and roles are operationalised in the healthcare context. The aim of this study was to explore the experiences of early career clinical academic NMAHPs who have undertaken, or are undertaking, clinical academic master’s and doctoral studies in the United Kingdom.

**Methods:**

We conducted 17 in-depth semi-structured interviews with early career clinical academics which included; nurses, midwives and allied health professionals. The data were analysed using thematic analysis.

**Results:**

Two themes emerged from the data; identity transformation and operationalising transformation. Both these highlighted the challenges and opportunities that early clinical academic training provided to the individual and organisation in which they practiced. This required the reconceptualization of this training from the pure acquisition of skills to one of personal and professional transformation. The findings suggest that individuals, funders, and organisations may need to relinquish the notion that training is purely or largely a transactional exchange in order to establish collaborative initiatives.

**Conclusion:**

Stakeholders need to recognise that a cultural shift about the purposes of research training from a transactional to transformative approaches is required to facilitate the development of NMAHPS clinical academics, to enable them to contribute to innovative health and patient care.

**Supplementary Information:**

The online version contains supplementary material available at 10.1186/s12909-020-02348-2.

## Background

Research is a core function of the National Health Service (NHS) provision, enabling improvements in the current and future health of the people it serves [1]. There is a growing body of evidence to suggest that research active healthcare organisations provide higher quality care, increased treatment choices and better clinical outcomes [2]. This requires a highly skilled and educated clinical workforce [3]. In 2007, the UK Clinical Research Collaboration [4] identified the need to develop capacity and capability in nurses, midwives and allied health professionals (NMAHPs) to develop clinical academic careers (CAC). Following these recommendations, funding was identified by Health Education England to support a fully funded non-medical clinical academic training pathway, administered by the National Institute for Health Research (NIHR) via its Academy. Initially this was awarded at Masters Level only, then further refined in a pathway that progressed from pre-masters internships to senior clinical lectureships [5]. Further guidance for organisations and staff has been developed by the Association of United Kingdom University Hospitals [6] and NIHR [7] to support the actualisation of clinical academic roles with the potential to transform patient care, outcomes and experiences.

Effective implementation of this framework requires clinicians to simultaneously develop into autonomous researchers leading, delivering, interpreting and contributing to clinical research whilst continuing to evolve clinical practice and clinical expertise [7]. There is emerging evidence regarding the internal and external infrastructures required for successful implementation [8–10]. Key success factors include: strategically aligned and collaborative partnerships; transformational clinical and research leadership; effective talent management strategies and targeted funding [11–13]. This is further emphasised in Richardson et al’s [14] examination of the career progression of NMAHPs pursuing independent research and clinical academic careers, where securing funding, experience and skills gained through training or research, and advice, support and guidance were identified as key enablers. In addition, nearly 50% of respondents reported inadequate support from their employing organisation as a barrier to their careers.

A qualitative exploration of CACs for NMAHPs [15] considered the role of educationalists in raising the profile of CACs. Key contributions included supporting the ongoing development of individuals and identifying and developing funding streams and career prospects in partnership with NHS organisations. Whilst this study provided useful insights into CAC development, it did not seek to capture the experiences of those undergoing research training or pursuing roles in this field. Meanwhile research examining the experiences of clinical research nurses, also identified the need for better integration between educational and practice organisations [8]. However, clinical research nurses predominantly support the delivery of research trials rather than designing and leading on programmes of research as aspired to by the NIHR vision [16]. Strategic commitment has been given to developing nursing and midwifery leadership in research within the ‘NIHR 70@70’ programme, providing targeted funding for activity to raise the profile of nursing research and opportunities for clinical academic career expansion [17].

Opportunities to develop clinical academic careers for NMAHPs via a specified pathway are now fairly well established, however evidence suggests that the ability for these pathways to be fully operationalised has been limited and do not mirror those afforded to medical graduates [18]. Locally collected evaluation data into the experiences of individuals on a bespoke CAC training programme [19] suggested that whilst some were able to pursue doctoral studies others merely returned to their clinical posts. Of the 32 NMAHPs students who undertook the training programme, 10 secured competitive peer reviewed funding for doctoral level study and 7 masters to PhD bridging awards. Anecdotally CAC research students report: altered perspectives on the relationship between evidence based practice and patient care; a recognition of the epistemological challenges of both clinical practice and research; as well as the practical demands of implementing these skills on their return to work.

While the structural obstacles to a fully functioning CAC may be gaining clarity, there remains an absence of empirical evidence on the personal and professional experiences of the transition from clinician or academic to clinical academic. Role conflict, challenges to identity and ethical dilemmas have been identified previously in dual roles such as lecturer-practitioner or practitioner-researcher [20–22]. Clinical academic roles have the potential to significantly impact on the quality of patient care provision, to inform efficient and effective service delivery, and we argue that their development requires increased prominence in healthcare, higher education and research [7, 23, 24].

In contrast, opportunities for CACs in medicine and dentistry have received greater investment and focus. Since 2003, annual surveys on the numbers and nature of Clinical Academics in Medicine and Dentistry have established that clinical academics make up around 6% of the medical workforce [25]. The majority of medical/dentistry clinical academics have substantive contracts with universities and honorary NHS contracts, spending approximately 50% of their time delivering direct patient care. There is continued growth in medical clinical academic career training and posts across the CAC journey, from internships through to Clinical Academic Professorial posts [26]. The Association of UK University Hospitals (AUKUH) outlined ambitions for 1% of the NMAHP workforce to hold clinical academic roles by 2030 [23]. However, the current clinical academic workforce in NMAHPs is estimated to be 0.1% despite [6] the development of training programmes and the potential contribution that NMAHP make to health and social care.

The aim of this study was to explore the experiences of early career clinical academic NMAHPs who have undertaken, or are undertaking, clinical academic masters and doctoral studies in the United Kingdom. By capturing these experiences we aimed to inform healthcare services and clinical academic programme providers on the key factors for a successful environment that best capitalises on their new expertise, passion and career aspirations.

## Methods

The study was informed by a critical theory and a realist epistemology, using qualitative semi-structured interviews to capture individual and shared experiences of early CAC training and its personal and professional impact [27].

Critical Theory takes as its starting point the empirical claim that our current (Capitalist) society is organised in a way that is unjust and fails to put emphasis on the power relations that serve to hinder progressive ideals [28]. As such the current cultural context encourages and normalises the ongoing intellectual development of CACs in medical and dentistry, but less so for NMAHPs. For NMAHPs the emphasis has been on practice and skills development across both academic and healthcare organisations. Critical Theorists take the view, that within a Capitalist economy, relationships tend to get turned into commodities with transactional value instead of having intrinsic value themselves. Treating education and research as a commodity, that is useful for transactional purposes only, is a danger. Transactional relationships are all about what you (the individual or institution) can get, and not about what you can give.

For the purposes of this study therefore we take as a starting point, that the failure to provide NMAHPs with institutional support to effectively evaluate and contribute to research about their own practice derives from a tendency for research training to be seen in transactional terms. This has contributed to both the disadvantaging of these professions, and has denied health service and patients the possibility of organising and delivering services using the insights, skills and contributions of these professional groupings. There has been a systematic privileging of medical knowledge and organisational principles that we argue may not always be in the best interests of patients and our health service. Developing CACs for these professional groups is therefore essential and more focus on how to better support them beyond research training is required. This study was designed to contribute to this.

### Sampling and recruitment

A purposive sample [29] of current and previous students of a nationally funded Masters in Research (MRes) programme at a Russell Group University was recruited using a maximum variation sampling approach. The aim was to recruit a minimum of six participants into each cohort; Current MRes (CM), MRes Alumni (MA) and current Doctoral Level Training (PhD). Participants were recruited on the basis of their role as a student on this pathway of CAC training at the Higher Education Institution (HEI). The maximum variation approach also took into account specific characteristics including profession and gender which was recorded from the participant demographic sheet. Sampling and data collection ran concurrently where sampling was informed by data collection and analysis, and further sampling was ceased once data saturation was reached where no new themes emerged [30].

An initial letter and participant information sheet was sent by the Chief Investigators (CD/AE) via email to current and alumni MRes students informing them of the study. Permission to be contacted by the Principal Investigators (AC/JC) was indicated by return email to the PIs or telephoning them, stating their preferred method of communication. The PIs met or discussed entry into the study via phone with potential participants. It was explained that entry into the study was entirely voluntary and that they could withdraw at any time. Informed written consent was obtained prior to data collection.

### Data collection

Interviews were carried out by two female clinical researchers (AC/JC), experienced qualitative researchers in healthcare and education. AC and JC were known to some participants through existing clinical networks. Participants completed a form outlining basic demographic data prior to the interview. All data were pseudo-anonymised; participants were given a study ID number which was kept in a secure, password protected drive.

Face to face interviews were conducted with participants according to their preferred location; home or private interview rooms at either their usual place of work or the HEI. A lone worker policy was followed. A provisional interview guide (see supplementary file) was devised informed by experience of the research team, literature on clinical academic role development, HEI clinical academic frameworks and career development tools [31, 32]. Questions were piloted with an MRes alumna and a clinical academic to ensure they were well framed and relevant. No changes were required. Interviews were audio recorded and transcribed verbatim.

### Data analysis

Data collection and analysis proceeded concurrently using thematic analysis “a method for identifying, analysing and reporting data” [33]. Data were independently analysed by AC and JC and involved six stages: familiarisation with the data (stage 1) were followed by identification of ‘themes’ (stage 2). Transcripts were then re-read and interpretative analysis conducted to create sub-themes (stage 3) subsequently refined into a finalised list of themes (stage 4 and 5). These were approved by the CIs investigator (CD/AE), to enhance rigour [34]. The final stage (stage 6) of interpretation involved discussing the themes relative to the existing evidence base and the research question.

### Reflexivity and rigour

The authors of this study are two Directors of the MRes programme (CD, AE), an ex MRes student (AC), and the local Trust Director for Research, Innovation and Professional Regulation (JC). We recognised that we are all invested in the success of this programme of study, and the development of CACs. To this end, transcripts were actively analysed for deviant cases [35] such as expectations and experiences that did not concur with the majority to promote dependability of the data and particularly those who did not want to pursue further identity transformation [36]. Interview transcripts were analysed separately by AC/JC to maximise transparency, accuracy and concordance when developing themes. Prior to further discussion and presentation of the analysis with other members of the research team, transcripts were anonymised to remove identifiable information. An in-depth description of the research analysis process, in addition to a reflective diary, was maintained to promote transparency of the data collection and analysis and later transferability of the findings.

### Ethical considerations

Ethical approval was sought and gained from the Medical School Research Ethics Committee (REC), School of Health Sciences, the University of Nottingham.

## Results

Seventeen participants (4 male, 13 female) with a mean age 38 years (range 26–55) were recruited to the study. There were seven nurses, five physiotherapists, two midwives, two occupational therapists and one mental health nurse. Participants had been qualified for a mean period of 11 years (range 4 to 24 years). The study achieved anticipated sampling of six participants in all but one group. This was however not deemed to impact negatively on the saturation of findings. Six participants were currently registered on the MRes, five had completed the MRes and returned to clinical practice and six participants were completing competitive peer reviewed PhD fellowships (see Table 1).

**Table 1:** Participant demographics

**Age (years)**	Range = 26–55 Mean = 38
**Sex**	Female = 13 Male = 4
**Time qualified (years)**	Range = 4–24 Mean = 11
**Profession**	Nurse = 8 Midwife = 2 Physiotherapist = 5 Occupational Therapist = 2
**Career stage**	Current Masters in Research Student = 6 Masters in Research Alumni (returned to clinical practice) = 5 Master in Research Alumni & current PhD Student = 6
**Total Participants**	17

The interviews lasted between 35 and 58 min. Two over-arching themes emerged from the findings; identity transformation and operationalising transformation. Both of these highlighted the challenges and opportunities that early CAC training provided the individual and organisation in which they practiced. Our data suggests that there is a need to reconceptualise this training opportunity away from a purely (commodified) transactional process of knowledge provision and acquisition, to one of personal and professional transformation. Both themes are now described in more detail using sample excerpts from transcripts and identifying participant group according to Current Masters (CM), Masters in Research Alumni (MA) and Masters in Research Current Alumni and current PhD (PhD).

### Theme one: identity transformation

This theme described how participants experienced changes to their clinical and personal identity. Participants explained how taking part in research training as aspiring clinical academics exposed them to theoretical concepts that underpinned knowledge generation thereby allowing them to, *“open their eyes to new ways of seeing the world … like walking into a new world.*” (CM2).

Research training was usually delivered in a multidisciplinary and multi-professional environment; contact with other students and academics outside the health and social care context exposed them to a wide variety of professions and academic disciplines. This allowed them to view their clinical environment and evidence based practice through a new ‘lens’. “*I think it was really good and healthy for us to do modules with other disciplines because health is so blinkered … The NHS is so huge and there are so many people in health we don’t have to look outside … but working with sociologists, economists gives you a different perspective.” (PhD4).*

Participants described an appreciation of how their new knowledge and emerging criticality could impact on their generation, understanding and application of evidence based practice on their immediate and future clinical academic practice. “*My previous experience in research had all been quantitative so it was good to get that breadth of different skills. Clinically it’s all about outcome measures, targets, so that opportunity to think about patient experience, how things affect people on a personal level and treating them as people, not numbers.” (MA3).*

However, this also challenged their perceptions of self and academic ability, with many of them having come from a clinical environment where they were experts in their field. They spoke of the difficulties they faced in embracing new academic concepts and how they felt out of their comfort zone, away from familiar clinical environments. “*I have loved it but it was a lot harder than I expected in terms of academic content, learning new languages. I can remember sitting in philosophy thinking, ‘I have absolutely no idea what you are talking about’! It’s been a real rollercoaster of emotions.” (CM2).* Whilst some participants found being exposed to new concepts enjoyable and stimulating, others failed to see the value of this to their clinical practice and NHS roles. *“I didn’t like the psychology and philosophy modules, they took me out of my box. I much preferred the clinically applied health parts.*” (MA1). This, on occasions led to disengagement with the training programme.

The movement from the clinical practice setting to a level four academic environment, combined with newly acquired academic knowledge and skills resulted in a transformation of how they viewed themselves. Key personal attributes such as resilience, tenacity and the ability to accept and act on criticism were frequently spoken of by participants. “*Resilience is the biggest one, pick yourself up. Knock backs are frequent and also the academic world is very different to the clinical world which you learn slowly. Critical appraisal, criticism at times is quite blunt, so you have to be quite thick skinned and in a clinical world it's sometimes wrapped in cotton wool.” (PhD3).* Participants also spoke of how undertaking the MRes provided opportunities for them to challenge previously held beliefs, views and experiences of how evidence based medicine is generated and applied in clinical practice. *“I hadn’t realised until now how much it has changed me. Looking at things from a wider perspective and I didn’t think I was particularly a judgemental person before I came on the [MRes] but it’s not until I have started thinking about things in a different way that I realised how presumptuous I was, how much I assumed without knowing. I have looked at my whole little bubble differently, the news, work, the world.” (CM2*).

Whilst participants commonly described undergoing a transformative experience, on return to practice, they were sometimes challenged by clinical peers and colleagues when attempting to implement their new found identify and knowledge. *“Care for improving patients experience is still at your heart and you need to show this because there is that perception that you have jumped ship and you don’t really care about patients anymore.” (PhD3).* Participants described the conflict and struggles they faced in discovering their new identity as a clinical academic NMAHP which was sometimes at odds with their previous clinical identity.

There was an acknowledgement that undertaking CAC training was not purely transactional but part of a transformational process that would require consideration of how it would then be operationalised on an individual, professional and organisational basis. *“For me the MRes was about gaining new research skills to bring to my NHS role, but it’s been so much more. It changed the way I think about and see my practice.”* (MA5).

### Theme two: Operationalising transformation

The second theme described the challenges and opportunities that aspiring clinical academics faced in operationalising their newly acquired skills and beliefs. Returning to practice, individuals were able to reflect on their previous clinical practice and that of others. They sought out opportunities: to critique existing practice; to consider how practices and services could be changed and evaluated; and to disseminate this and influence others. *“Before I would read new articles on practice and theory and try new things with patients that would be at an appropriate level, but now I would be able to assess with reasonable accuracy as to what may or may not work, and how the research would translate across to my patients and their conditions. But also sharing these findings with my group of clinicians.” (CM4).* There was recognition that implementing newly acquired skills, developing capacity and capability in others and considering how to actualise a CAC required careful consideration of how they might be perceived by others. *“You have to be quite politically aware, you’ve got to be the right sort of character with the knowledge because it’s quite powerful and it could also be destructive if it was applied wrongly … .these careers are new, you have been given a very prestigious insight into how research is conducted and how you go about creating new knowledge that probably a lot of others have not got, so you have to be politically aware when you review current practice. For example it would be no good going into a clinical area and refusing to work there because care there is not underpinned by high quality evidence.” (PhD2).* Other participants talked of the struggles they faced when trying to embed their new skills and identity into their NHS organisations, who were perceived to have differing priorities. *“Strategically they [managers] value CAC but practically they don’t know what to do with me.”* (PhD1). This led to frustration amongst some participants and a sense that their training and skills were not valued.

Participants explained how early career clinical academic training was emancipatory and shaped the direction of their career considerations and trajectories. Participants spoke of actively seeking new opportunities which allowed them to utilise their new academic and personal skills. “*When I went back [to work] I spent 2-3 days seeing patients and then I was looking for a project so I got involved in a project so pulling data together analysing and presenting the data so all the skills I had learned at university for a service bid.” (MA1).* They highlighted how they viewed the world of clinical practice differently, not only identifying problems but now being skilled in how to answer them and approaching them with a newfound appreciation for critical enquiry. This required negotiation with the organisation to support them and provide opportunities to use their skills to evaluate current and future practice. For some individuals there was still a perceived value in undertaking the MRes even if they decided not to pursue a further doctoral level study. *“I don’t want to do a PhD … but when I went for a project manager job, they asked questions about data analysis, audit and having done the MRes, having that on my CV and application I think helped me get the job.”* (MA2).

Other participants were clear that the training inspired them to seek funding and prospects for doctoral level studies. However, being successful required organisational support, and where opportunities were not available, they started to look elsewhere. “*I had been back at work for 2 months before I started looking for new jobs. I probably would have done that anyway but the MRes opened up so many more doors for me. So before I was only looking at band 7 [profession] job but after the MRes I started looking at research jobs, how I could apply for a PhD.” (MA1).*

Participants spoke of the many challenges they faced operationalising a clinical academic career; these were primarily concerned with concurrent or sequential environmental change. “*There is no career path. You have to carve out that role for yourself, to look for the opportunities, use your contacts. Sell myself and say this is good for me and good for you. No-one is going to come along and say here you go.” (CM5).*

Participants were cognizant of the need to identify individuals and networks of influence that could support them in their personal development but work with them to act as advocates for change. They highlighted that whilst there was a defined CAC pathway, they were also agents for defining what individual clinical academic careers could look like in practice. However, this required openness on their behalf to achieve individual and organisational change. *“On the MRes they have that lovely painted clinical academic career pathway of doing the MRes, getting a fellowship, a post-doc and that’s all lovely if you can do that but doesn’t always happen so you have to be flexible.” (PhD3).* This continued struggle for further funded PhD fellowships and opportunities to apply their newly acquired skills led some participants to give up on their CAC ambitions and return to previous roles. *“Post MRes I did want to do more research. I did secure funding for one day a week for a year from the trust to recruit more patients for my study as I didn’t get enough patients recruited so it allowed me to continue my project. But the funding ran out and it’s sat on my to do pile ever since. I have never got round to finishing it and it’s been about two years now.” (MA3).*

Access to mentors and role models was described as critical to realising their clinical academic ambitions and implementing their newly found skills and knowledge. Having visionary leaders who could see the value of investing in clinical academic careers and aspiring clinical academic NMAHPs was frequently said to help participants, *“Having leaders who can help you pull things together and see the bigger picture.”* (PhD4).

## Discussion

This study is, to the authors knowledge the first to explore the experiences of those undertaking non-medical early clinical academic research training. It highlights how what is initially perceived to be a transactional process of undertaking research methods training and development becomes a process of transformation for the individual, their profession and organisation. In doing so it exemplifies how the initial value of the commodity, i.e. the research training, may initially be conceived as having transactional value, is then replaced by a recognition that this is a mechanism to facilitate the transformation into critical thinking and a research identity suitable for transforming professional selves and elements of practice. However, this has the potential to create dissonance that requires flexibility from all health and social care stakeholders to create a ‘receptive organisational culture’ for CAC’s ‘previously recognised by Gerrish et al. [11].

A central theme from the findings was that participants learnt to see themselves and their professional practice differently, as a result of moving out of practice and accessing training in research methods. Education has had a long history associated with providing people with knowledge and understanding to enable citizens to actively participate in a democratic society [37, 38]. Previous qualitative research into the transition from clinician to academic has suggested that this is a process that requires a shift in the culture of the organisation as well as the identity of the individual, and can take up to 3 years [39]. Undertaking CAC training provides opportunities to make a contribution to the provision of health and social research and clinical practice that extends beyond the pure acquisition of research methods skills. This study suggests that being able to realise the full benefits of individual and organisational transformation requires strong collaborations between HEIs and health and social care providers, supporting previous commentary [11, 14].

Traditional non-medical career pathways post qualification have focussed predominantly on clinical, managerial and academic (including lecturer/practitioner) roles within nursing, midwifery and allied health professionals. Clinical academic careers therefore represent a disruption from the expected career trajectory. Individuals’ career biographies were transformed as a result of clinical academic training that exposed them to new and diverse concepts, ideas, experiences and knowledge. Participants in this study spoke of this experience as emancipatory although many faced challenges in developing and operationalising this emerging identity within their healthcare organisations. Working in organisations and systems with traditional role and career structures led to conflict and frustration but was also perceived to provide a wealth of opportunities to challenge the status quo, carving out new roles to deliver and influence patient care. The emancipatory possibilities for NMAHPs both in terms of their own professional practice, and the care provided to patients within an increasingly commodified healthcare environment is considerable if CACs are more effectively supported [40].

The concept of biographical disruption was first described by Bury [41] in relation to chronic illness and how this impacts on individuals’ identity and perception of self. Biographical disruption is shaped by more than just an individual’s experience, identity and beliefs, because it’s a process that inculcates socio-cultural, political and professional debates and power struggles [42, 43]. By building a new ‘clinical academic’ biography for themselves, participants began to develop a new identity and some found it easier than others to thrive and embed the new identity within clinical practice. This study found that whilst some individuals were able to thrive and transcend the biographical disruption, others struggled to make sense of their new identity in a largely transactional healthcare environment. Transactional organisations are largely based on the premise of successful completion of tasks, where leaders focus on specific goals and objectives, followed by the use of rewards or reprimands [44]. In many healthcare settings, daily work and tasks are designed and implemented around transactional models which do not foster innovation, challenge or deviation from the norm. The transformed identities of individuals described in this study found the transactional processes, roles and boundaries of healthcare frustrating and these were frequently cited as the main barriers to realising their clinical academic ambitions. As a greater number of healthcare providers move towards transformational models of leadership and operationalisation, aspiring clinical academics are likely to thrive in these environments and challenge some of the ways in which healthcare may not be in patient’s interests.

### Strengths and limitations of study

It is, to the authors knowledge, the first time that experiences of non-medical pre-doctoral clinical academic training has been explored. The strengths of the study are that a broad and representative sample was recruited to the study, reflecting the range of professionals and genders. Semi-structured interviews with experienced qualitative researchers allowed the phenomena of clinical academic training, careers and individual experiences to be explored. Members of the study team were known to a number of participants and this may have facilitated recruitment and data collection. As local and national advocates of CACs they have situational knowledge of training for NMAHP clinical academics and the challenges they face in practice. Prior knowledge of study area is considered by some as an essential pre-requisite for situated understanding and positive action [45]. Reflexive diaries kept by the team were used to critique the position of the researcher and their social interaction with participants [46, 47]. Consideration of these suggested shared knowledge of the study context, enhancing dialogue, data analysis and the overall validity of the study findings.

The main weakness of the study is that the majority of the participants undertaking doctoral level training had not yet returned to clinical practice (and so were still training) so their experiences of operationalising CAC could not be explored in terms of their longer term career trajectory. It is recommended that future research should explore the long term impact of clinical academic career training from an individual and organisational perspective in order to fully explore the agenda and provide robust support and guidance. It is recognised that this study only explored clinical academic training provided a Russell Group University offering NIHR Clinical Academic Training and that these opportunities and findings may not be transferable to other higher education institutions where this is not the case. The insider position of some members of the study team has been highlighted; it could be suggested that this could inhibit the disclosure of sensitive information, and lead to shared assumptions about knowledge generation. However, critical reflection throughout data collection and analysis, the use of research diaries, combined with secondary validation of thematic analysis, and the development of a theoretical explanatory framework suggest transparency, credibility and trustworthiness of findings was established [48].

## Conclusion

There remains uncertainty around role definitions and expectations of NMAHP clinical academics. In order to realise the benefits of the growing clinical academic workforce, funded training opportunities and the recognition of this as a discrete career pathway are needed [7]. Individuals, funders, and organisations may need to relinquish previous identities to establish collaborative initiatives to meet the 2030 clinical academic workforce targets [23]. Stakeholders need to recognise that a cultural shift from transactional to transformative approaches is required to facilitate the development of NMAHPS clinical academics, to enable them to contribute to innovative health and patient care, as has been spearheaded by the medical and dentistry workforce. This has the potential to facilitate NMAHPs contribution to leading programmes of research which seek to enhance safe, patient centred, clinically effective and cost-effective healthcare provision.

## Supplementary Information

**Additional file 1.**

## Data Availability

The datasets used and analysed during the current study are available from the corresponding author on reasonable request.

## Abbreviations

AUKUH: Association of UK University Hospitals
CAC: Clinical Academic Careers
CI: Chief Investigator
CM: Current MRes
HEI: Higher Education Institution
MA: MRes Alumni
MRes: Masters in Research Methods
NHS: National Health Service
NIHR: National Institute for Health Research
NMAHPs: Nurses, Midwives and Allied Health Professionals
PI: Principal Investigator
REC: Research Ethics Committee
