# Supplementary material for: Capitalising on the transformational opportunities of early clinical academic career training for nurses, midwives and allied health professionals
Source: BMC Med Educ. 2020 Nov 10;20:418. doi: 10.1186/s12909-020-02348-2 (PMC7653772; doi:10.1186/s12909-020-02348-2)
Supplement: Supplementary file 1 — Additional file 1. [file 12909_2020_2348_MOESM1_ESM.docx]

**Interview Schedule**

**Cohort 1 – Current MRes Students**

How did you first find out about the MRes?

What was your clinical role when you applied for the MRes?

Can you tell me what led you to apply for the MRes?

What support did you receive during your application?

What do you expect to get from completing the MRes

What are your experiences of the MRes so far?

What are your perceptions of CAC’s?

What were your thoughts on pursuing a CAC/PhD when you have finished?

How do you think the MRes will help equip you for a CAC?

How do you think the MRes will impact on your clinical practice?

What do you think the challenges are to achieving a CAC?

What do you think the enablers are to achieving a CAC?

Do you think you will attend conferences as a result of your MResstudies?

Are you planning to publish your dissertation findings?

**Cohorts 2 and 3 – MRes Alumnae and PhD Students**

Can you tell me what led you to apply for the MRes?

What were your experiences of undertaking the MRes?

What support did you receive during your MRes?

Prompts: organisation, personal, family/home situation

What were your thoughts on pursuing a CAC when you finished?

When you started the MRes were you intending to pursue a PhD: never? Immediately? After you’d returned to practice to consolidate what you had learnt?

What impact has the MRes had on your

1. Clinical practice
2. Clinical Academic career
3. Future studies

What did you expect the MARes to deliver?

What do you think the challenges are to achieving a CAC?

What do you think the enablers are to achieving a CAC?

What are your aspirations for your career?

Have you attended conferences as a result of your MARes studies?

Have you or are you planning to publish your dissertation findings?
